# Emerging Stronger from the COVID-19 Crisis: Recover, Reposition, and Redesign

**DOI:** 10.1089/pop.2020.0188

**Published:** 2020-10-05

**Authors:** Andrew Waldeck, Prashant Srivastava, Harold A. Picken

**Affiliations:** ^1^ Innosight Consulting, Lexington, Massachusetts, USA.; ^2^ Huron Consulting Group, Inc., Chicago, Illinois, USA.

**Keywords:** COVID-19 recovery, crisis response, disruption, health care transformation, recover-reposition-redesign

In 6 months, COVID-19 has wreaked havoc on the US health care system and economy. As of July, the United States reported more than 3 million COVID-19 cases and more than 130,000 deaths.^1^ Real gross domestic product declined at a rate of 4.8% in the first quarter (after growing by 2.1% in the fourth quarter of 2019).^2^ In April, more than 20 million Americans reported losing their jobs. Across the country, infection rates are increasing, and hospital and other resources are under significant strain.

As devastating as these effects are, there is reason for guarded optimism. “Big Event” disruptions like the pandemic present incredible challenges, but also offer unique opportunities. With an understanding of history, health care leaders can navigate near-term uncertainty while accelerating needed transformation. By doing so, the ultimate impact of COVID-19 will be defined not by the aforementioned metrics but by its role in driving 3 changes: (1) compelling provider organizations to act more as a “system,” (2) transforming the care model to achieve the Quadruple Aim,^3^ and (3) ensuring that care is organized around the consumer.

## Understanding Big Events

Our team has analyzed past Big Event disruptions such as the 1918–1919 and 1957–1958 influenza pandemics, economic shocks such as the Great Depression and global financial crisis of 2008, and the September 11, 2001 terrorist attacks.^4^ By looking across these Big Events, we have found 3 key principles to follow.

### Principle #1: there is a silver lining

Despite having a near-term negative impact, Big Events create conditions favorable to new growth. During the 1957–1958 influenza pandemic, Sony introduced its transistor radio into the United States. The product sold more than 7 million units, created a new industry category, and accelerated Japan's economy for the next decade. Businesses facing greater financial and operating pressure focus more intently on what customers want and innovate to address unmet needs. Consumers, facing their own constraints, are forced to experiment with new ways of solving important needs. Following the 2008 financial crisis, many small businesses looked for alternative sources of capital. Square, the payments start-up, tapped into this need and found quick success. Today, Square, Airbnb, and other businesses created during the financial crisis have created an estimated $250B of value.

To capture the silver lining, health care leaders need to adopt a growth mind-set. Early in the pandemic, providers accumulated a large backlog of deferred appointments, referrals, and procedures. Recapturing this backlog will require addressing consumers' fears concerning safety and developing a trusted relationship with them. This provides an opportunity for systems to make strides in innovating consumer experience and differentiating from competitors to capture market share. Many systems are adopting a centrally coordinated approach to offer consumer-friendly hours and the ability to schedule appointments at the nearest location. To alleviate infection concerns, systems are aggressively communicating efforts to ensure patient safety. Some systems are creating “COVID clinics” to consolidate volume or creating stand-alone wards within existing facilities. Finally, there has been tremendous growth in virtual visits, accelerated by growing consumer demand, increasing physician adoption, and enabling government regulation.

### Principle #2: not all behaviors are created equal

Big Events lead to changes in individual behavior – sometimes modest, sometimes more substantial. In 2011 Londoners faced a 2-week strike that closed Underground stations, forcing them to experiment with new commuting patterns. Post strike, 5% of commuters stuck with their new routines. In contrast, airline volume did not return to pre-9/11 levels until 2005, changing the industry more permanently.

An important and largely unanswerable question surrounding Big Events is whether these shifts will be temporary or permanent. Consider the explosive growth in virtual health. Jefferson's JeffConnect telemedicine platform grew from ∼50 to ∼2500 virtual visits per day in March. Many systems now must understand how much volume to expect from this new delivery channel. The answer depends on whether observed behaviors by physicians, consumers, and payors will persist.

Instead of trying to predict which of these new behaviors become habits, leaders need to answer a different question. Which of these new behaviors do they want to encourage to stick? Promoting desirable behaviors will require systems to understand an individual's needs and to identify what levers to pull to influence that individual's behavior.

For example, many systems are accelerating decision-making through the creation of centralized “Command Centers,” dedicated units that track real-time intelligence, establish daily critical activities, and oversee rapid progress. These centers demonstrate the value of certain behaviors: focusing on a limited set of clear priorities, communicating frequently, and increasing data transparency. Additionally, many of our clients are seeing greater collaboration across parts of the organization where, historically, there has been friction.

Many leaders are concerned that this progress will subside as the pandemic passes. At one health care system, executives wanted to adjust their operating model to reinforce improvements in rapid decision-making. The system started by documenting new behaviors such as being more collaborative, acting decisively, and taking greater ownership, and tried to identify their root causes: smaller, focused teams with communications and administrative support, new data-visualization techniques, and delegated authority. To reinforce these behaviors, leadership eliminated an entire management committee and replaced it with the expectation that accountable leaders will gather as needed to resolve issues.

### Principle #3: be bold: recover, reposition, and redesign

Because Big Events change behavior, systems' response strategies must seek to do more than just recover. The goal is not to return to how things used to operate. With certain habits being relaxed, this creates a window of opportunity to make bold changes before individuals settle into new routines.

Consider Adobe. The software maker long wrestled with the decision to move to a Software-as-a-Service (SaaS) model. During the 2008 financial crisis, it saw favorable conditions for action and made a substantial acquisition to move entirely to SaaS. Over the next decade Adobe transitioned from its largely product sales model to a consumer-centric model with constant customer connectivity and predictable recurring revenues. As a result, Adobe has tripled in size and its stock price appreciated at almost 30% per year.

The implication for health care leaders is clear. Beyond employing crisis management techniques to stabilize near-term finances and operations, leaders must determine how to *reposition* existing capabilities to maximize near-term resilience while also *redesigning* the system to drive long-term sustainability. Some systems are exploring transformative mergers and acquisitions, while others are looking to reshape their market footprint, scope of services, workforce composition, and real estate portfolios.

**FIG. 1.**
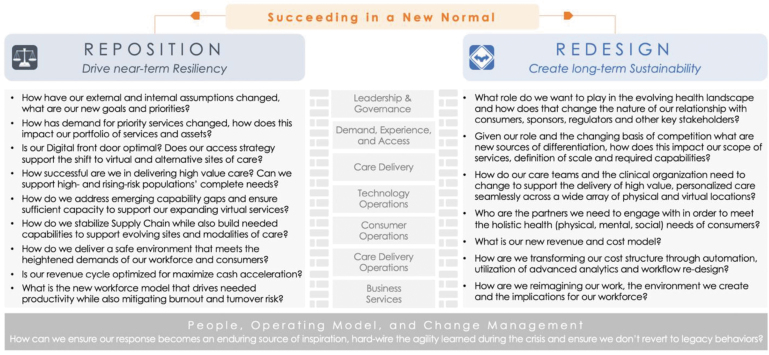
Succeeding in a new normal.

Figure 1, created by the authors, outlines the key reposition and redesign considerations for health systems.

## The Impact of Big Events on Health Care

### Acting more as a system

“Systemness” is an organizational and managerial approach that emphasizes the quality and efficiency of the whole rather than the performance of each of the parts. Providers have struggled to act as single systems, particularly ones that have grown through acquisition. This has resulted in component organizations competing with each other and decision-making inefficiencies. Moreover, incentives and other operating model elements reinforce a focus on the “part” versus the “whole.”

A “systemness” approach produced pronounced benefits for Jefferson Health during the pandemic response. The system recognized the value of enterprise-level planning while dealing with urgent challenges of acquiring medical supplies, personal protective equipment, and backfilling exposed staff. “Our whole is quite literally greater than the sum of our parts. Leveraging the resources and talents of Jefferson's 14-hospital health system and its university have maximized our overall response to the communities that we serve.”^5^

Likewise, during recovery, systems will need to centralize decision-making and coordination to deal with increased financial and operational pressure, and ensure consistent quality and experience across digital and analog touch points. For example, throughput management should be applied consistently across the system to ensure effective social distancing during scheduled visits. Systems need to be able to flexibly shift capacity across facilities. Finally, virtual care requires system-level investment, shared technical infrastructure, and coordination on issues such as safety and experience.

### Transforming the care model

The term “care model” defines the who, what, where, and how for health services that are delivered to a given individual, population, or patient cohort. Systems have made notable progress in changing where and how care is delivered by driving incremental shifts to lower intensity settings and utilizing a wider array of caregivers. Virtual health has accelerated this trend and created potential for greater convenience, reduced costs, and better utilization of physical capacity. Ambulatory models such as CVS Health's HealthHubs, Walgreens partnership with VillageMD, and Walmart Health's Clinics will likely only grow. COVID-19 also has strained health care workers, and care models must incorporate more flexible work arrangements and expand available resources.

A significant opportunity for systems is to reconfigure their services – for example, focusing more on behavioral health. The Kaiser Foundation reported that nearly half of Americans feel COVID-19 has negatively impacted mental health.^6^ In addition, COVID-19 casts greater light on racial and ethnic health disparities. African Americans account for more than 30% of COVID hospitalizations yet represent 18% of the population studied.^7^ Health systems have the opportunity to advance their portfolio and address these issues by closing the digital divide for those at socioeconomic risk, developing new personalized care models, and impacting broader health determinants through community partnerships.

### Organizing around the consumer

Organizations with a strong consumer capability deeply understand an individual and what drives behavior. While there is progress in this area, providers tend to have an inward perspective: organizing around specialties and locations where services are delivered. The fee-for-service economic model drives attention around the type of insurance consumers have rather than an understanding of an individual's needs and challenges.

The pandemic has the potential to accelerate the move to the consumer because it underscores the importance of behaviors occurring outside the boundaries of health systems. Some 60% of people's health is determined by the choices they make and where they live.^8^ During the pandemic, many consumers delayed critical health care procedures out of concern for personal safety. Emergency department visits declined by more than 40%.^9^ Necessary treatments for conditions such as diabetes, kidney disease, and cancer were impacted significantly. With COVID-19, the decisions of individuals directly affect the overall health of populations and the resulting challenges facing health systems. For example, the individual decision to wear a mask and socially distance directly affects the severity of the surge health systems have to manage and whether elective procedures would be further affected.

**FIG. 2.**
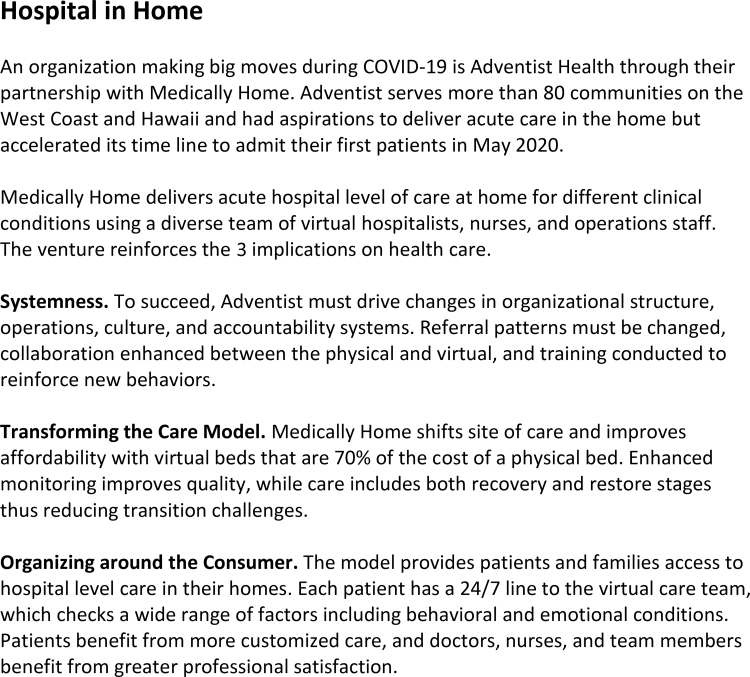
Hospital in home.

Figure 2 highlights these concepts using a real-world example.

## Conclusion

Big Event disruptions provide a unique window of opportunity for leaders to take bold steps to ensure near-term resiliency and long-term sustainability. The hospital in home model is emblematic of how health systems should consider big strategic moves to drive shifts toward systemness, consumer-centricity, and care transformation. We hope that these principles serve as inspiration for leaders to drive growth and accelerate health care's transformation.
